# Identification of Dobrava-Belgrade Virus in *Apodemus flavicollis* from North-Eastern Italy during Enhanced Mortality

**DOI:** 10.3390/v14061241

**Published:** 2022-06-07

**Authors:** Stefania Leopardi, Petra Drzewnioková, Melissa Baggieri, Antonella Marchi, Paola Bucci, Marco Bregoli, Paola De Benedictis, Federica Gobbo, Laura Bellinati, Carlo Citterio, Isabella Monne, Ambra Pastori, Gianpiero Zamperin, Elisa Palumbo, Francesca Festa, Martina Castellan, Maira Zorzan, Emilio D’Ugo, Paolo Zucca, Calogero Terregino, Fabio Magurano

**Affiliations:** 1 OIE Collaborating Centre for Diseases at the Animal-Human Interface, Istituto Zooprofilattico Sperimentale delle Venezie, 35020 Legnaro, Italy; sleopardi@izsvenezie.it (S.L.); pdrzewniokova@izsvenezie.it (P.D.); mbregoli@izsvenezie.it (M.B.); pdebenedictis@izsvenezie.it (P.D.B.); fgobbo@izsvenezie.it (F.G.); lbellinati@izsvenezie.it (L.B.); ccitterio@izsvenezie.it (C.C.); imonne@izsvenezie.it (I.M.); apastori@izsvenezie.it (A.P.); gzamperin@izsvenezie.it (G.Z.); epalumbo@izsvenezie.it (E.P.); ffesta@izsvenezie.it (F.F.); mcastellan@izsvenezie.it (M.C.); mzorzan@izsvenezie.it (M.Z.); 2 Department of Infectious Diseases, Istituto Superiore di Sanità, 00161 Rome, Italy; melissa.baggieri@iss.it (M.B.); antonella.marchi@iss.it (A.M.); paola.bucci@iss.it (P.B.); emilio.dugo@iss.it (E.D.); fabio.magurano@iss.it (F.M.); 3 Biocrime Center, Central Directorate for Health, Social Policies and Disability, 34127 Trieste, Italy; zucca.paolo@regione.fvg.it

**Keywords:** Hantavirus, Dobrava-Belgrade, *Apodemus flavicollis*, one health

## Abstract

Hantaviruses include several zoonotic pathogens that cause different syndromes in humans, with mortality rates ranging from 12 to 40%. Most commonly, humans get infected through the inhalation of aerosols or dust particles contaminated with virus-containing rodent excreta. Hantaviruses are specifically associated with the host species, and human cases depend on the presence and the dynamics of reservoir hosts. In this letter, we report the identification of Dobrava-Belgrade virus (DOBV) in the yellow-necked mouse (*Apodemus flavicollis*) from Italy. The virus was detected in the mountainous area of the province of Udine, bordering Austria and Slovenia, during an event of enhanced mortality in wild mice and voles. Despite serological evidence in rodents and humans that suggested the circulation of hantaviruses in Italy since 2000, this is the first virological confirmation of the infection. Phylogenetic analyses across the whole genome of the two detected viruses confirmed the host-specificity of DOBV sub-species and showed the highest identity with viruses identified in Slovenia and Croatia from both *A. flavicollis* and humans, with no signs of reassortment. These findings highlight the need for ecologists, veterinarians and medical doctors to come together in a coordinated approach in full compliance with the One Health concept.

## 1. Introduction

Hantaviruses are enveloped viruses belonging to the family Hantaviridae with segmented, negative sense, single-stranded RNA. The genus Orthohantavirus includes 23 species classified by the International Commission on the Taxonomy of Viruses (ICTV), plus about 30 other viruses for which a complete characterization is not available yet [1]. In contrast to other viruses of this family, hantaviruses are transmitted directly from animal reservoirs with no arthropod vectors involved.

Most hantaviruses have been described in rodents (order Rodentia, families Cricetidae, Muridae, Nesomyidae and Sciuridae) and new species are increasingly found in bats (order Chiroptera) and insectivores (order Soricomorpha) [2]. Each species of Hantavirus seems to be specifically associated with its own reservoir host. In Europe, the main reservoir species are the wild yellow-necked mouse (*Apodemus flavicollis*), the black-striped field mouse (*Apodemus agrarius*) and the bank vole (*Myodes glareolus*), maintaining two different strains of the Dobrava-Belgrade virus (DOBV) and Puumala virus (PUUV), respectively. In addition, the grey rat (*Rattus norvegicus*) potentially maintains Seoul virus (SEOV) globally, sporadically reported outside Asia, including a single identification in France [3].

Hantaviruses, particularly the several species associated with rodents, are known as zoonotic pathogens that cause acute febrile diseases in humans with a mortality rate ranging from 12% for haemorrhagic fever with renal syndrome (HFRS) to 40% hantavirus cardiopulmonary syndrome (HCPS). Although most cases are reported from China, Europe notifies more than 9000 severe cases each year, with several others likely remaining undetected [4,5]. These cases are mainly related to Puumala and Dobrava-Belgrade viruses, with mortality usually lower than 1% and ranging from 5 to 15%, respectively [3,4,6]. As humans are exposed mostly by inhaling viral particles aerosolized from the saliva, urine and/or faeces of rodents, occupations and activities such as working in forestry, farming and road maintenance strongly increase the likelihood of viral infection [5]. In addition, the incidence of human cases seems to be highly correlated with the dynamics of the populations of the reservoir species [6]. In particular, the massive increase in population density caused by an abundance of food during the mast years of beech (*Fagus sylvatica*) and oak (*Quercus robur*) trees can enhance the circulation of hantaviruses in the wild during the following year and increase the risk of human exposure [6,7,8,9]. The fact that mast-seeding events are linked to environmental factors, such as higher temperatures and seasonal changes in rainfall, suggests that the burden of hantavirus disease might increase as a consequence of climate change [10]. In this context, there is a major need for an integrated approach between ecologists, veterinarians and medical doctors, in full compliance with the One Health concept [11,12].

In Italy, only eight cases of Hantavirus disease have been described, mostly from tourists or transboundary workers, suggesting they contracted the infection abroad. Among these, most had a clinical presentation of HFRS, but three cases related to infection with the new world hantavirus Sin Nombre virus, showed typical signs of HCPS [5]. To date, no native cases of hantavirus disease have been described in Italy, despite the proximity to endemic countries and the presence of both the wild yellow-necked mouse, the black-striped field mouse and the bank vole [5]. However, antibodies neutralizing either PUVV or DOBV have been historically detected in both humans and rodents, particularly in the mountainous areas bordering Austria and Slovenia, suggesting that these pathogens are actively circulating at least in some parts of the country [5,12,13]. The present communication reports the first molecular finding of *Dobrava-Belgrade orthohantavirus* in *Apodemus flavicollis* from north-eastern Italy in Summer 2021.

## 2. Materials and Methods

Between June and July 2021, the diagnostic laboratories at the Istituto Zooprofilattico Sperimentale delle Venezie (IZSVe) received 35 rodent carcasses from the mountainous area bordering Austria and Slovenia, in the province of Udine, that were necropsied. Twenty-one samples consisting of pools of liver and lungs from one to five individuals were sent to the Department of Infectious Disease (IDD) at the Italian National Institute of Health (ISS) for molecular detection. Further analysis was conducted at the IZSVe on individual kidney samples to determine the percentage of positive samples using a nested RT-PCR [14] and to confirm the host species through sequencing of the cytochrome oxidase I, as described elsewhere [15]. All samples positive for the detection of *Orthohantavirus* spp. were initially characterized through Sanger sequencing before attempting whole genome sequencing of viruses from individual samples. No further analyses were performed on the pooled samples, because they included more than one positive individual that could have challenged correct reconstruction of the sequences. We used a target approach amplifying the three segments using four pairs (one for S, one for M and two for L) of DOBV specific primers modified from Taylor et al., [16] (Table 1). The reaction was performed in a two-step RT-PCR reaction using SuperScript™ IV Reverse Transcriptase (Invitrogen, Waltham, MA, USA) and Platinum™ SuperFi II Green PCR Master Mix (Invitrogen). Positive samples were sequenced using next generation sequencing implemented in Illumina MiSeq with Reagent Nano Kit v2 (500 cycles). 

To obtain complete genomes for the viruses, we first assessed the quality of raw reads using FastQC v0.11.7 (https://www.bioinformatics.babraham.ac.uk/projects/fastqc/, accessed on 26 January 2022) and removed the reads related to the Illumina Nextera XT adaptors (Illumina, San Diego, CA, USA) using scythe v0.991 (https://github.com/vsbuffalo/scythe, accessed on 26 January 2022). We then used cutadapt v2.10 to trim the adaptors, designed specifically for Hantavirus, and to filter the raw reads with a Q score below 30 and a length below 80 nucleotides [17]. Complete genomes were generated through a reference-based approach using BWA v0.7.12 (https://github.com/lh3/bwa, accessed on 26 January 2022) [18]. We then used loFreq v2.1.2 (https://github.com/CSB5/lofreq, accessed on 26 January 2022) [19] to call single nucleotide polymorphisms (SNPs) and applied an in-house script to obtain the consensus sequences, setting 10× as the minimum coverage and 50% of allele frequency as the threshold for base calling.

Viral sequences obtained in the study were aligned with reference strains using G-INS-1 and default parameters implemented in Mafft [20]. Maximum likelihood (ML) nucleotide phylogenetic trees were inferred using PhyML (version 3.0) implemented in Seaview employing the GTR+Γ4 substitution model, a heuristic SPR branch-swapping algorithm and 100 bootstrap reiterations [21]; the obtained trees were edited online for graphical display using iTOL (version 6.0) [22].

## 3. Results

Between June and July 2021, the diagnostic laboratories at the Istituto Zooprofilattico Sperimentale delle Venezie (IZSVe) received 35 rodent carcasses collected from the mountainous area bordering Austria and Slovenia, in the province of Udine. Necropsies showed no significant macroscopic lesions. Genetic analyses classified the hosts as *Apodemus flavicollis* (n = 22) or *Myodes glareolus* (n = 8), whereas five analyses provided no interpretable sequences, likely due to the advanced decay stage of the carcasses. Among the twenty-one liver-lung pools, two tested positive for *Orthohantavirus* spp. Further analysis conducted on individual kidney samples confirmed the positivity of four individuals of *A. flavicollis*, with a percentage of positivity of 11.7% in the yellow-necked mice. All voles tested negative. Sanger sequencing of the PCR product attributed the infection to Dobrava-Belgrade virus. 

**Figure 1:**
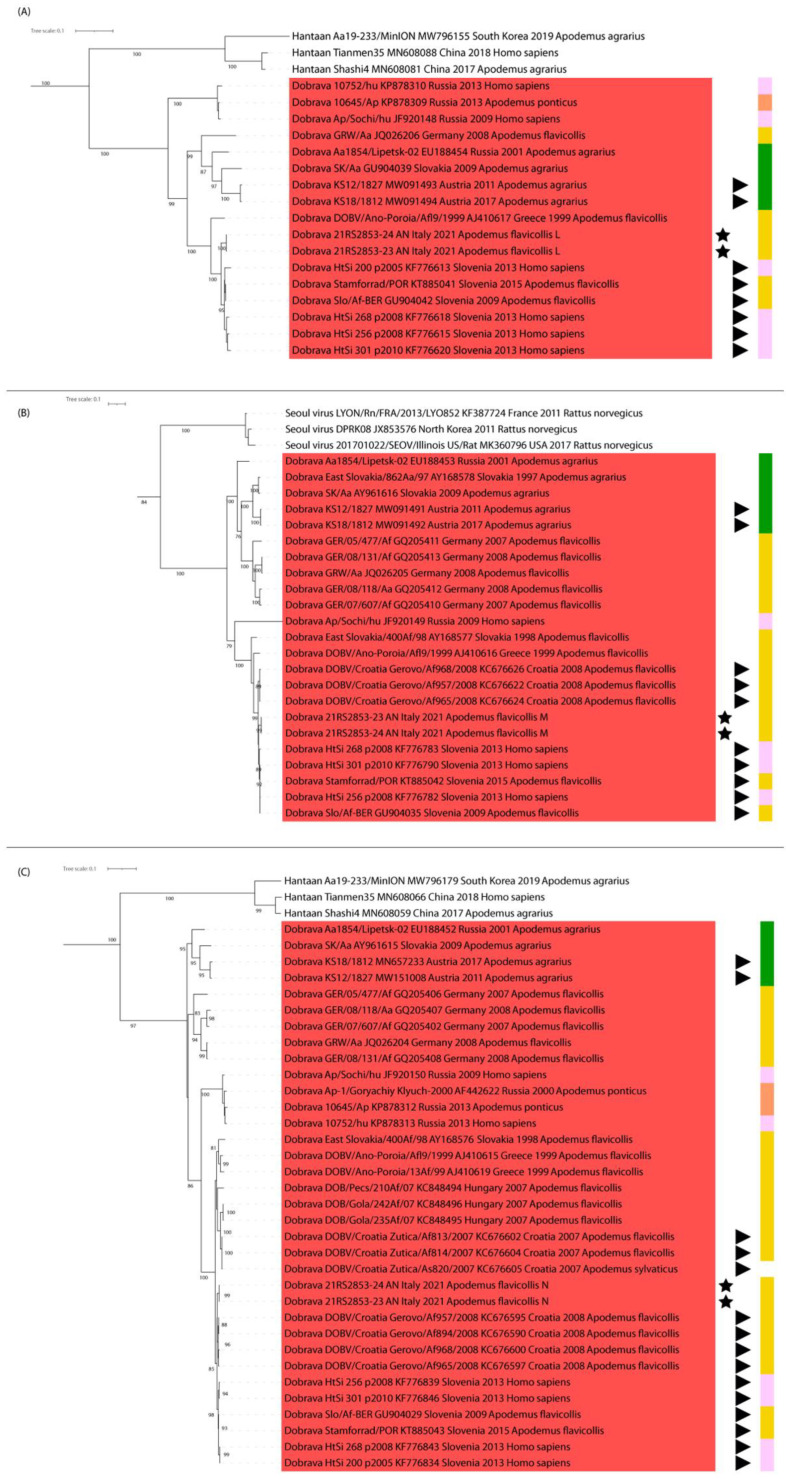
ML phylogenetic trees of the whole sequences for segments L (**A**), M (**B**) and S (**C**). The red box shows the viruses belonging to the specie *Dobrava-Belgrade orthohantavirus*. Original sequences are marked with a star, and sequences from countries neighbouring the study area are indicated with arrows. Coloured boxes represent the host in which the strains were detected, namely *A. flavicollis* (yellow), *A. ponticus* (orange), *A. agrarius* (green) and human infections (pink).

Target next generation sequencing performed from individual samples provided the complete genomes of two viruses, with an average coverage ranging respectively between 2967× and 3152×. All consensus sequences were submitted to GenBank under accessions n. OM677634-OM677639, raw data were submitted to SRA with accession n. PRJNA807277, and BioSamples SAMN25965836 and SAMN25965837. Maximum likelihood phylogenetic reconstructions using whole genome sequences demonstrated that the Italian DOBV clustered within variants found in *A. flavicollis* and humans from Croatia and Slovenia in all three segments (Figure 1A–C) and showed no signs of genetic reassortment. Highest identity was found with strains from *A. flavicollis* in Slovenia (SLO/Af BER, with an identity of 97.5% in L and 97.8% in M) and Croatia (Croatia_Gerovo/Af968/2008, with an identity of 98.7% in S and no sequences available for the remaining segments). 

## 4. Discussion and Conclusions

The fact that hantaviruses are specifically associated with a certain host species makes the presence of these reservoirs the most relevant risk factor for human infection. Other than the grey rat, all rodents known as hantavirus reservoirs in Europe are present in north-eastern Italy, including both *A. flavicollis* and *A. agrarius*, that have been found to maintain different sub-species of *Dobrava-Belgrade orthohantavirus* [23]. In this short report, we describe the identification of DOBV in the yellow-necked mouse from the Italian mountainous area bordering Austria and Slovenia. Phylogenetic analyses confirmed that our sequences grouped only with variants from the yellow-necked mouse, confirming the host-specificity of DOBV sub-species [23]. Despite serological evidence in rodents and humans the suggested the circulation of hantaviruses in Italy since 2000, this is the first virological confirmation of the infection [5,13,24]. 

Previous studies questioned why hantaviruses should be restricted to a limited portion of the distribution of their hosts [25]. Indeed, our findings suggest that negative findings in certain areas could be due to a lack of or ineffective surveillance rather than the true absence of the pathogen. Indeed, the number of rodents received from north-eastern Italy in 2021 is the highest reported from the laboratory over the past 5 years, with average submissions between 2017 and 2020 being about 15 individuals with 1 single sample analysed from the province of Udine before 2021. Such an increase in rodent diagnostic activity was associated with enhanced mortality reported from north-eastern Italy. In Italy, previous efforts in active surveillance were pivotal in supporting the circulation of both DOBV and PUVV using serology but failed to identify the virus for over 20 years. This is not new in the study of pathogens in their wild reservoir, where they often circulate at low prevalence, making serology the most effective tool for monitoring the infection in the absence of viral detection [26]. Periodic epidemic waves have been largely described for both PUVV and DOBV in the case of relevant variations in population dynamics [6,7,8,27]. In our case, positive findings could have been favoured by increased prevalence within the reservoir due to increased population density, likely associated with the high production of seed in 2020, reported as a mast year across Europe [28]. This hypothesis is consistent with the enhanced mortality of rodents experienced in the area that was likely associated with exceeding the carrying capacity for the species rather than to the viral infection. However, it is crucial to notice that all the samples collected in 2012 after a similar event tested negative, which further highlights the challenges related to the study of pathogens in wildlife and might suggest changes in the ecology of hantaviruses in Italy that can be only unravelled through longitudinal active and passive surveillance. In addition, genetic data from our investigation show that the increased mortality was not peculiar to mice but also involved voles, despite the absence of virus detected in *M. glareoulus*, the reservoir species for PUVV. Since serological evidence suggests that PUVV might circulate in the country as well [13,24], our negative result could indicate that the sample size of bank voles was too low to detect the virus, or it could be related to differences in the dynamics of the two viral species in the area. 

Numerous studies have revealed that outbreaks of hantavirus disease in humans are often associated with a high prevalence of the pathogen within the reservoir [4,11,12]. In this context, Slovenia reported several cases of PUVV over spring-summer in 2021 and compatible syndromes were also recorded in the provinces of Trieste and Udine, with a single case confirmed by molecular approaches that had contracted the infection in neighbouring Slovenia. Our study, together with previous serological evidence [5,12,13], confirms the circulation of DOBV in the wild yellow-necked mice in north-eastern Italy. Data presented herein encourage both active and passive surveillance plans in wild mice, taking into account population dynamics to better understand hantavirus ecology in the areas bordering their putative distribution, with the final aim of mitigating the risk of infection in humans. Indeed, this first identification in the reservoir emphasizes the risk of occurrence in humans of mild and severe forms of hantavirus disease in Italy, HFRS and HCPS included. It also reveals the need to conduct further research on hantaviruses in Italy, and to increase the awareness of physicians and local populations regarding the transmission pathways of the viruses and the available diagnostic methods. Equally important is the need to implement and integrate effective surveillance systems.

## Figures and Tables

**Table 1:** Primers used for targetted amplification of the complete genome of DOBV.

Segment	Primer Orientation	Sequence (5′→3′)	bp
S	Sense	TAGTAGTAKRCTCCCTAAARAGCACTAYAC	1673
	Antisense	TAGTAGTAGRCTCCCTAAAAAGACATTCAGGAAGC	
M	Sense	TAGTAGTAGRCTCCGCAAGAAAYAG	3664
	Antisense	TAGTAGTAKGCTCCGCARGATATAG	
L1	Sense	TAGTAGTAGACTCCGGAAGAGACARAYTAC	3253
	Antisense	CATYCCKACACCRAAAAGAGATGAAC	
L2	Sense	GATAACTCAGCTAARTTYAGAAGRTTCAC	3351
	Antisense	TAGTAGTATGCTCCGGAAAATGAAAATRAAT	

## Data Availability

All consensus sequences are deposited in GenBank under accessions n. OM677634-OM677639, and raw data can be found in SRA with accession n. PRJNA807277, BioSamples SAMN25965836 and SAMN25965837.
